# Astrocyte-derived adenosine is central to the hypnogenic effect of glucose

**DOI:** 10.1038/srep19107

**Published:** 2016-01-12

**Authors:** Emeric Scharbarg, Marion Daenens, Frédéric Lemaître, Hélène Geoffroy, Manon Guille-Collignon, Thierry Gallopin, Armelle Rancillac

**Affiliations:** 1 Brain Plasticity Unit, ESPCI-ParisTech, PSL Research University, Paris, F-75005, France; 2 CNRS, UMR 8249, Paris, F-75005, France; 3 Ecole Normale Supérieure-PSL Research University, Département de Chimie, Sorbonne Universités - UPMC Univ Paris 06, Paris, F-75005, France; 4 CNRS, UMR 8640 Pasteur, Paris, F-75005, France

## Abstract

Sleep has been hypothesised to maintain a close relationship with metabolism. Here we focus on the brain structure that triggers slow-wave sleep, the ventrolateral preoptic nucleus (VLPO), to explore the cellular and molecular signalling pathways recruited by an increase in glucose concentration. We used infrared videomicroscopy on *ex vivo* brain slices to establish that glucose induces vasodilations specifically in the VLPO via the astrocytic release of adenosine. Real-time detection by *in situ* purine biosensors further revealed that the adenosine level doubles in response to glucose, and triples during the wakefulness period. Finally, patch-clamp recordings uncovered the depolarizing effect of adenosine and its A_2A_ receptor agonist, CGS-21680, on sleep-promoting VLPO neurons. Altogether, our results provide new insights into the metabolically driven release of adenosine. We hypothesise that adenosine adjusts the local energy supply to local neuronal activity in response to glucose. This pathway could contribute to sleep-wake transition and sleep intensity.

Sleep disorders occur worldwide and are comorbid with numerous pathologies including obesity, diabetes, heart attack, depression and stroke. Although our understanding of the physiological mechanisms of sleep induction and maintenance are far from complete, several key aspects regarding wakefulness have been resolved. Specifically, this tightly regulated state of consciousness results from a combination of drives, among which the homeostatic sleep drive is one of the strongest^1^. Indeed, cerebrospinal fluid contains several sleep-promoting factors that accumulate during wakefulness including adenosine (ADO), which is considered to be the most potent. This endogenous sleep-promoting factor modulates the duration and intensity of slow-wave sleep (SWS)^2^,^3^. Only two out of the four adenosine receptor subtypes (A_1_, A_2A_, A_2B_ and A_3_) have been shown to mediate the sleep-inducing effects of adenosine: the inhibitory G protein-coupled adenosine A_1_ receptor (A_1_R) and the excitatory G protein-coupled adenosine A_2A_ receptor (A_2A_R)^3^. In the ventrolateral preoptic nucleus (VLPO), which contains the GABAergic and galaninergic neurons responsible for SWS onset and maintenance^4^,^5^,^6^, ADO acting through A_1_R is proposed to indirectly promote sleep via the disinhibition of sleep-active neurons, whereas activation of A_2A_R could directly stimulate these neurons^7^,^8^,^9^. However, proof for the direct activation of sleep-promoting neurons by ADO is still debated^10^,^11^.

One energy hypothesis of sleep has posited that the function of sleep is to restore brain energy metabolism, with extracellular ADO levels allowing the brain to assess the need for sleep^12^. Indeed, energy stores decrease during the metabolically active waking period and are restored during sleep^12^,^13^. Although the function of sleep in regulating energy metabolism seems more complex than initially proposed^14^,^15^, wakefulness in rodents is known to be reduced after a meal and to increase during fasting^16^. Moreover, glucose is reported *ex vivo* to directly inhibit the neuronal firing rate of orexinergic neurons that are responsible for wake promotion^17^ and, conversely, to excite VLPO sleep-promoting neurons identified according to their inhibitory response to bath-applied noradrenalin (NA)^8^,^18^,^19^,^20^. By monitoring the gating of their ATP-sensitive potassium channels (K_ATP_) these sleep-active neurons adapt their firing frequency according to the extracellular glucose concentration^21^. In contrast, neighbouring neurons depolarized by NA are glucose-insensitive^21^. Furthermore, bilateral microinjection *in vivo* of glucose into the VLPO of mice revealed an increase in SWS, associated with an increase in c-Fos expression specifically in this region^21^. Altogether, these results strengthen the link between sleep processes and the extracellular energy status.

The neuro-glio-vascular network is a complex mechanism that allows the brain to adjust local blood flow to neuronal needs. To better understand the cellular and molecular mechanisms triggered by glucose, we have now recorded *ex vivo* the vascular response to an increase in extracellular glucose concentration in mouse VLPO slices. By monitoring blood vessel diameter changes as the output of local network activity, we have established that glucose induces vasodilation specifically in the core of the VLPO, via the astrocytic release of ADO. These results could have broad implications ranging from adapting our meal composition according to our sleep needs, to the prevention of diseases related to sleep deficits.

## Results

### Glucose-induced vasodilation is specific to the VLPO core

**Figure 1:**
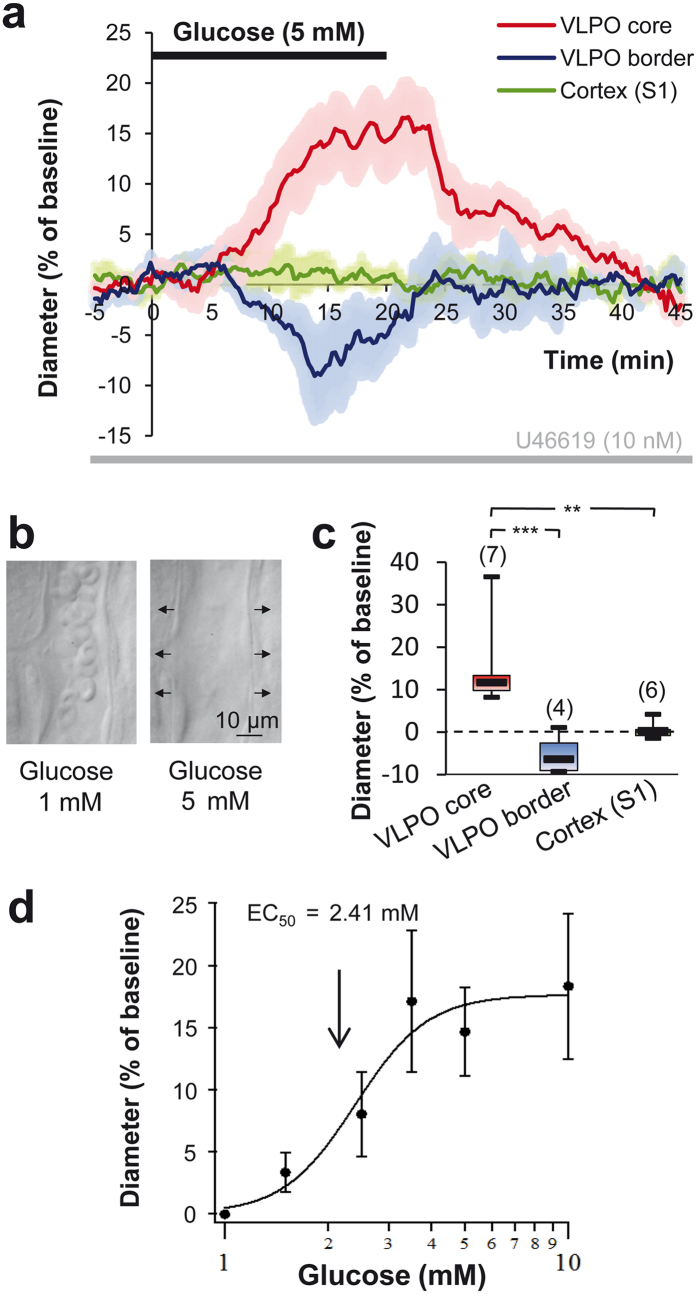
VLPO arterioles are glucose sensitive. (**a**) Mean ± SEM vascular responses induced by the extracellular increase in glucose concentration from 1 to 5 mM in the VLPO core (in red; *n* = 7), as compared to the VLPO border (in blue; *n* = 4) and to the somatosensory cortex (S1) (in gree*n*; *n* = 6) in vessels preconstricted with U46619. (**b**) Photomicrograph of an arteriole dilating in response to the increase in glucose concentration in the VLPO core. (**c**) Box plots of the vascular response measured at 19–20 min after the glucose switch in the VLPO core, at its border and in the somatosensory cortex (S1). The median is plotted as the central black bar of the box, the upper and lower quartiles are the edges of the box and the whiskers show the extremes of the distribution. Sample sizes are indicated in parentheses. (**d**) Dose-response curve of the glucose effect. **P < 0.01, ***P < 0.001; Mann-Whitney *U*-test.

To investigate the functional role of glucose in the control of vascular tone in the VLPO, we recorded by infrared videomicroscopy the changes in blood vessel diameter following an increase in the extracellular glucose concentration, from 1 to 5 mM. These concentrations are consistent with physiological changes in glucose concentration in brain parenchyma^22^,^23^. Interestingly, we found that an increase in extracellular glucose concentration reversibly dilates intraparenchymal arterioles within the VLPO, to 14.68 ± 3.53% over the baseline (Wilcoxon test, *P* ≤ 0.001; *n* = 7; Fig. 1 and Video 1). The dose–response analysis of this glucose-induced vasodilation revealed an EC_50_ of 2.41 mM (Fig. 1d). Surprisingly, the increase in glucose-concentration induced an opposite effect on blood vessels located at the medial and lateral borders of the VLPO, where we observed a mean vasoconstriction of 3.39 ± 1.02% (Wilcoxon test, *P* < 0.05; *n* = 4; Fig. 1a,c). This penumbra induced by glucose could result from vascular steal^24^ or from a decrease in the neuronal activity due to local inhibition^25^,^26^, since sleep-promoting neurons are assumed to be GABAergic. Finally, we observed that blood vessel diameter in the somatosensory cortex (S1) was insensitive to the increased glucose concentration (0.80 ± 0.82%, Wilcoxon test, *P* = 0.492; *n* = 6; Fig. 1a,c), confirming that glucose only has a slight effect on cerebral blood flow regulation *in vivo*^27^. This region-specific effect of glucose in the VLPO *vs*. the somatosensory cortex could involve neuronal and/or glial signalling properties that differ between brain areas^28^. Indeed, region-specific regulation of protein expression and astroglial reactivity may be related with specific roles of neuroglial interactions and neuronal circuits regulation^29^,^30^.

### Adenosine is central to the glucose-induced vasodilation in the VLPO

**Figure 2:**
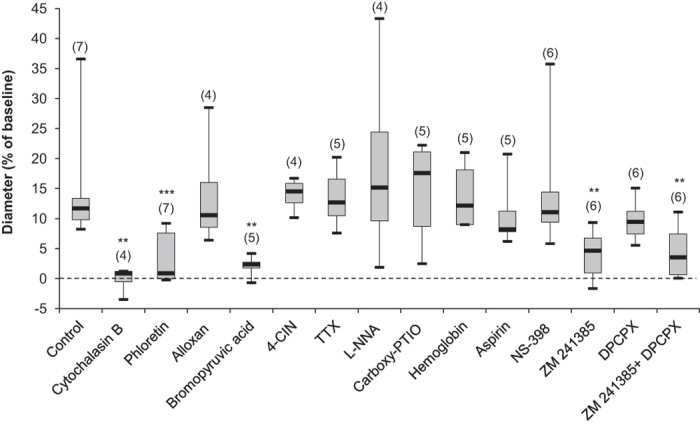
Pharmacological determination of the molecular pathways underlying glucose-induced vasodilation. Vasodilations induced by the increased glucose concentration from 1 to 5 mM were significantly altered in the presence of: a non-selective glucose transporter (GLUT) inhibitor, cytochalasin-B (Cyto B; 25 μM); phloretin (10 μM; a GLUT1 inhibitor); bromopyruvic acid (100 μM; a hexokinase inhibitor); and ZM 241385 (10 μM; a selective adenosine A_2A_ receptor antagonist). Alloxan (10 mM; an inhibitor of glucokinase), 4-CIN (500 μM, an inhibitor of lactate and pyruvate transport), TTX (1 μM; which blocks action potential discharge), L-NNA (100 μM; a nonspecific NOS inhibitor), carboxy-PTIO (30 μM; a specific nNOS inhibitor), haemoglobin (4 μM, a NO scavenger), aspirin (50 μM; a cyclooxygenase-1 (COX-1) inhibitor), NS-398 (10 μM; a selective (COX-2) inhibitor) and DPCPX (10 μM; a selective A_1_ adenosine receptor antagonist) had no significant effect on glucose-induced vasodilation. The median is plotted as the central black bar of the box, the upper and lower quartiles are the edges of the box and the whiskers show the extremes of the distribution. Sample sizes are indicated in parentheses. **P < 0.01, ***P < 0.001, for pharmacological compounds *vs*. control condition in 5 mM glucose alone; Mann-Whitney *U*-test.

The cellular and molecular pathways underlying the glucose-induced vasodilation in the core of the VLPO were assessed with different pharmacological treatments (Fig. 2). First, we found that in the presence of the non-selective glucose transporter (GLUT) inhibitor cytochalasin B (25 μM), glucose-induced vasodilation was significantly impaired (−1.15 ± 1.01%; Mann-Whitney *U*-test, *P* < 0.006; *n* = 4), suggesting that glucose uptake into the intracellular compartment is required. Even in the presence of the GLUT1 inhibitor phloretin (10 μM), the vasodilatory response to glucose was still significantly impaired (3.57 ± 1.77%; *P* < 0.001; *n* = 7). This result suggests a major role for astrocytes in glucose uptake, since GLUT1 is predominantly expressed in the blood-brain barrier (including the astrocytes), whereas neurons mainly express GLUT3^31^. We next investigated whether glucose metabolism within the intracellular compartment is necessary for glucose-induced vasodilation using a nonspecific hexokinase inhibitor, bromopyruvic acid (100 μM). Glucose-induced vasodilation was significantly blocked (2.06 ± 0.89%; *P* < 0.003; *n* = 5), indicating that glucose must be metabolized within the cellular compartment. However, the specific inhibition of glucokinase (hexokinase IV) by alloxan (10 mM) did not alter the mean vascular response (13.99 ± 4.96%; *P* > 0.65; *n* = 4). Therefore, the observed glucose-induced vasodilation does not indicate any glucose metabolization by neurons, since glucokinase is selectively expressed in neurons^32^,^21^.

The putative involvement of neurons in glucose-induced vasodilation was also investigated by blocking the lactate shuttle. Vasodilation was unaltered in the presence of 4-CIN (500 μM), a specific inhibitor of the lactate transporter (13.98 ± 1.17%; *P* > 0.32; *n* = 4; Fig. 2). Finally, the contribution of vasoactive compound normally released in response to neuronal activity was assessed by preventing discharged action potentials with TTX (1 μM). This treatment did not affect the vasodilatory response to glucose (13.51 ± 2.49%; *P* > 0.755; *n* = 5), reinforcing the previous observations that there is a non-neuronal contribution to this mechanism.

To further investigate the molecular pathway mediating glucose-induced vasodilation, we examined the putative involvement of nitric oxide (NO), a potent vasodilator^33^,^34^. Lowering basal NO levels through treatment with the specific NO synthase (NOS) inhibitor L-NNA (100 μM; 18.88 ± 8.81%; *P* > 0.53; *n* = 4; Fig. 2) or with NOS scavengers such as carboxy-PTIO (30 μM; 14.42 ± 4.26%; *P* > 0.88; *n* = 5) and haemoglobin (4 μM; 13.85 ± 2.23%; *P* > 0.88; *n* = 5) did not significantly alter the glucose-induced vasodilations as compared to the control condition. Next, we assessed the involvement of prostaglandin, another important vasodilator, by inhibiting its synthesis. Neither aspirin (50 μM; 10.84 ± 2.91%; *P* > 0.20; *n* = 5; Fig. 2), a cyclooxygenase-1 (COX-1) inhibitor, nor NS-398 (10 μM; 14.68 ± 4.82%; *P* > 0.84; *n* = 6), a selective COX-2 inhibitor, were capable of blocking glucose-induced vasodilations.

Several reports in the literature have indicated that there is a significant sleep drive mediated by a glio-dependent accumulation of ADO^35^,^36^ that is also involved in the regulation of cerebral blood flow (CBF)^37^,^38^. We therefore investigated the putative involvement of this purine in the glucose-induced vasodilatory response. In the presence of a selective A_2A_R antagonist (ZM 241385; 10 μM), glucose-induced vasodilations were significantly reduced (4.01 ± 2.11%; *P* < 0.002; *n* = 6; Fig. 2). In contrast, the switch in glucose concentration did not induce any significant vasomotor changes in the presence of a selective A_1_R antagonist (DPCPX; 10 μM; 9.68 ± 1.53%; *P* > 0.30; *n* = 6). Moreover, no additive response could be observed in the presence of either adenosine receptor antagonist (4.55 ± 2.57%; *P* < 0.05; *n* = 6), suggesting that the vasodilatory response to glucose in the VLPO relies on ADO release and subsequent activation of A_2A_R but not A_1A_R. This result reinforces previous reports showing that adenosine applied externally to cerebral blood vessels induces vasodilations via A_2A_R located in brain blood vessels, most likely on endothelial cells^39^,^40^,^41^.

### Real-time measurements reveal ADO release in response to increased extracellular glucose concentration

**Figure 3:**
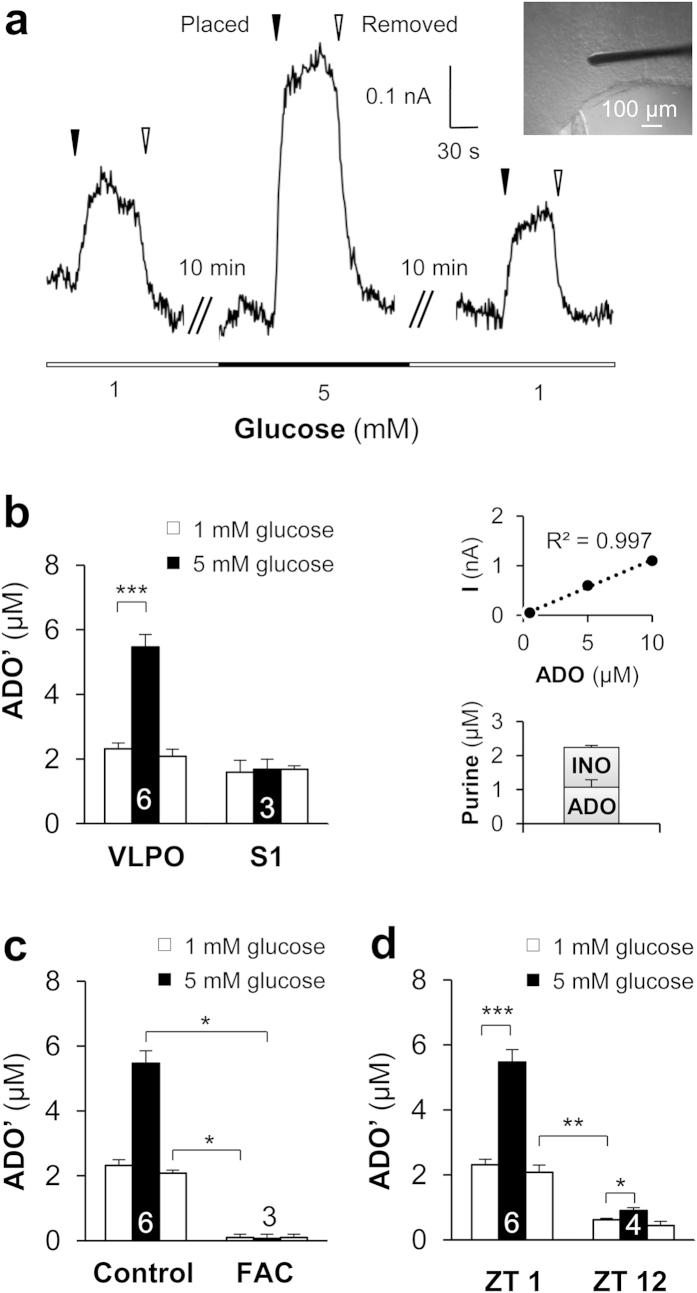
*In situ* measurements of adenosine by purine biosensors. (**a**) Representative recording of real-time ADO’ signals within the VLPO. ADO biosensors were lowered into the VLPO area (‘Placed’; black arrowheads) and raised (‘Revoved’ white arrowheads) successively in 1, 5 and 1 mM glucose to prevent the probe desensitization. Insert: Photomicrograph of an ADO biosensor within the VLPO. (**b**) Left: histogram comparing adenosine release in the VLPO and in the cortex following a rise in glucose concentration. Right: Example of a calibration curve from the adenosine biosensor showing a linear response from 1 to 10 μM adenosine (top), with ratios of ADO (*n* = 6) and INO (*n* = 3) detected in the VLPO (bottom). (**c**) The adenosine response is significantly impaired in the presence of a glial toxin. In the presence of fluoroacetate (FAC; 5 mM), adenosine levels remained below the detection limit for both 1 and 5 mM glucose. This suggests that adenosine tone is provided by the glial cell, in the form of additional adenosine released following the increased glucose concentration. (**d**) Adenosine response to glucose at the beginning and end of the mouse rest period. Slices performed at light onset (Zeitgeber time 1 [ZT 1] = 9 AM, *n* = 6) showed a significantly higher concentration of adenosine as compared to slices performed at the onset of mouse diurnal activity (dark period, [ZT 12], *n* = 4). In both cases, the increased glucose concentration enhanced the level of adenosine detected i*n situ* by ADO probes. *P < 0.05, **P < 0.01, ***P < 0.001; Mann-Whitney *U*-test.

In order to confirm that a rise in extracellular glucose concentration is associated with an increase in ADO released in the VLPO, we performed real-time measurements of ADO with purine enzymatic biosensors^42^,^43^,^44^. Measurements with ADO probes represent total purinergic responses and were referred to as ADO’. In the VLPO, ADO’ levels were initially 2.63 ± 0.17 μM in 1 mM glucose and significantly increased to 5.42 ± 0.36 μM in 5 mM glucose; ADO’ levels finally decreased to 2.31 ± 0.22 μM when the VLPO was returned to 1 mM glucose perfusion (*P* < 0.001; *n* = 6; Fig. 3a,b). In contrast, increased glucose concentration failed to induce any significant ADO release in the cortex (from 1.57 ± 0.31 μM to 1.68 ± 0.31 μM; *P* = 0.93; *n* = 3, Fig. 3b). These results support our previous pharmacological experiments, suggesting that the increase in glucose concentration induces an extracellular ADO release that is region-specific.

To further investigate whether ADO is released by astrocytes in response to glucose, we performed real-time measurements of ADO in slices that were pre-treated and continuously perfused with the glial toxin fluoroacetate (FAC; 5 mM). As shown in Fig. 3c, FAC treatment completely abolished the adenosinergic tone as well as the ADO response to increased glucose concentration (0.1 ± 0.1 μM; *P* < 0.05; *n* = 3). This result strengthens the role of astrocytes as essential effectors of the glucose-dependant increase in extracellular ADO levels.

To investigate the relationship between ADO release and the circadian cycle, measurements of ADO levels in low (1 mM) or high (5 mM) glucose concentration were performed both at Zeitgeber time 1 (ZT 1; 1 h after the start of the light cycle) and at ZT 12 (at the dark cycle onset). In 1 mM glucose, we found that the ADO level was significantly lower at ZT 12 (0.62 ± 0.04 μM; n = 4) than at ZT 1 (2.64 ± 0.17 μM; Mann-Whitney *U*-test, *P* = 0.019; *n* = 6; Fig. 3d). Nevertheless, the increase in glucose concentration at ZT 12 was still able to induce significant ADO’ release, even if this increase was weaker (48.44 ± 2.41%) than at ZT 1 (70.90 ± 6.87%). These results are the first *ex vivo* demonstration in VLPO slices that aspects of ADO release vary in parallel with circadian rhythms.

### Increased extracellular glucose differentially modulates VLPO neuronal activity

**Figure 4:**
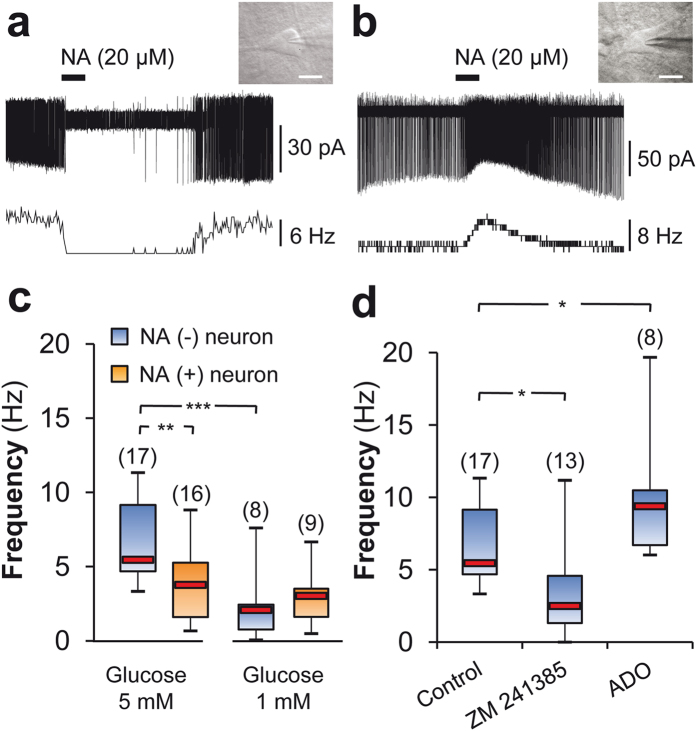
Glucose sensitivity of NA (−) neurons in the VLPO relies on adenosine release. (**a**,**b**) Reversible inhibitory (**a**) and excitatory (**b**) effects of bath-applied NA (20 μM) on the spontaneous firing activity of neurons referred to as NA (−) and NA (+), respectively. Recordings were performed in loose cell-attached configuration. The associated firing frequencies are represented in the lower trace of each figure. Inserts: Photomicrographs of the recorded neurons. Scale bar: 20 μm. (**c**) Box plots of NA (−) and NA (+) neuron firing frequencies in high (5 mM) and low (1 mM) glucose concentrations. (**d**) Box plots of NA (−) firing frequencies in 5 mM glucose, in the control condition, in ZM 241385 (A_2A_R antagonist; 10 μM), and after 1 min application of adenosine (ADO, 100 μM). The median is plotted as the central red bar of the box, the upper and lower quartiles are the edges of the box, and the whiskers show the extremes of the distribution. Sample sizes are indicated in parentheses. *P < 0.05, **P < 0.01, ***P < 0.001; Mann-Whitney *U*-test.

To decipher how astrocytic-derived ADO modulates the firing of VLPO neurons, we performed loose-cell attached patch recordings on neurons previously identified by their response to bath-applied noradrenalin (NA) (Fig. 4a,b). Putative sleep-active neurons are inhibited by this neurotransmitter (which is released during wakefulness^8^,^18^,^19^,^20^) and were assigned as NA (−) neurons; other VLPO cell types depolarized by NA were assigned as NA (+) neurons. We observed that the firing frequency was significantly decreased only in NA (−) neurons when the glucose concentration was lowered from 5 to 1 mM (6.56 ± 0.68 *vs.* 2.21 ± 0.91 Hz; Mann-Whitney *U-*test, *P* < 0.001; Fig. 4c). The putative contribution of A_2A_R activation in the glucose-induced excitation of VLPO neurons was assessed by adding the specific A_2A_R antagonist (ZM 241385, 10 μM). In 5 mM glucose, this treatment significantly decreased the spontaneous firing rate of NA (−) neurons to 4.13 ± 1.14 Hz (Mann-Whitney *U*-test, *P* < 0.05, Fig. 4d), revealing a tonic excitation of VLPO NA (−) neurons by A_2A_R at high glucose levels. In contrast, bath-applied ADO (100 μM) significantly increased the firing rate of NA (−) neurons (Fig. 4d). These results reinforce the hypothesis that ADO is able to promote the excitation of VLPO sleep-promoting neurons. Furthermore, we have demonstrated that ADO application continues to increase the firing rate of NA (−) neurons in 5 mM glucose, indicating that A_2A_R was not saturated in this condition.

Two different types of VLPO NA (−) neurons have been described according to their pharmacological response to serotonin (5-HT) in rats. Type-1 neurons are inhibited by 5-HT, whereas Type-2 neurons are excited by 5-HT^8^. Only Type-2 neurons are excited by ADO via the postsynaptic activation of A_2A_ adenosine receptors^8^. Therefore, we recorded the pharmacological response to 5-HT application in a subset of neurons whose firing rate was previously increased in response to ADO. We found no significant difference between Type-1 and -2 VLPO neurons, which respectively increased their firing frequencies by 69.23 ± 9.46% and 71.79 ± 2.21% on average (Mann-Whitney *U*-test; *P* > 0.685; *n* = 4 and *n* = 4) in response to bath application of ADO. Thus, these experiments indicate that in mice, VLPO Type-1 and -2 neurons are not distinguishable by their response to ADO.

### Adenosine depolarizes NA (−) neurons via A_2A_ receptors

**Figure 5:**
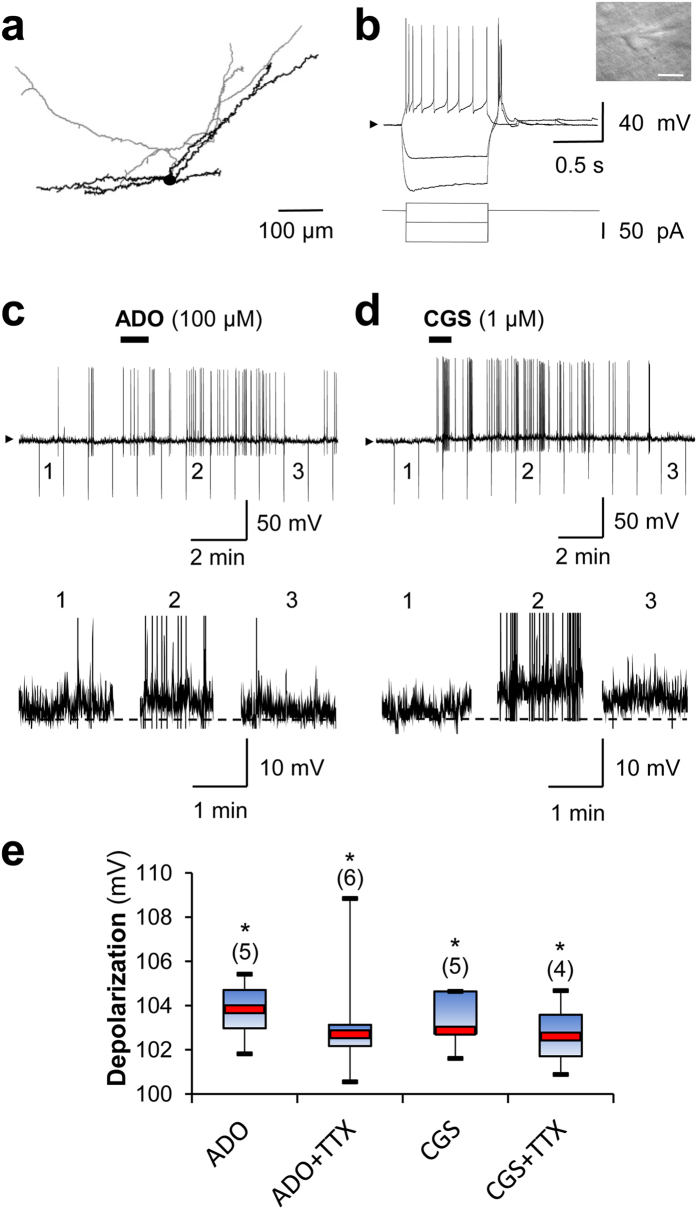
Adenosine depolarizes NA (−) neurons throughout A_2A_R activation in the VLPO. (**a**) Neurolucida reconstruction of a typical NA (−) neuron in the VLPO. Dendrites are represented in black and the axon is in grey. (**b**) Electrophysiological behaviour of the neuron shown in (a). Voltage responses are induced by current injections of −100, −40 and 20 pA from a holding potential of −60 mV (arrow head). Insert: infrared image of the recorded neuron. Scale bar: 20 µm. (**c**) Current-clamp recording of the neuronal depolarization induced by bath application of ADO (1 min) in a NA (−) neuron from a holding potential of −56 mV (arrow head, top panel). The recorded membrane potential is shown at higher magnification in the lower trace. (**d**) Current-clamp recording as in (c), except with an A_2A_R agonist application (CGS, 1 min). (**e**) Box plots of the depolarizing effects of ADO, ADO+TTX, CGS and CGS+TTX. The median is plotted as the central red bar of the box, the upper and lower quartiles are the edges of the box, and the whiskers show the extremes of the distribution. Sample sizes are indicated in parentheses. *P < 0.05; paired *t*-test.

The excitatory effect of ADO on NA (−) neurons was further investigated in whole-cell current-clamp recordings at 1 mM glucose (Fig. 5). Cells were filled with biocytin during recordings and were subsequently stained at the end of the experiment to confirm their neuronal localizations within the VLPO (Fig. 5a). Bath application of ADO (100 μM) significantly depolarized NA (−) neurons by 3.75 ± 0.93 mV (paired *t*-test, *P* = 0.013; *n* = 5; Fig. 5c,e), even under TTX (1 μM; 3.34 ± 1.43 mV; paired *t*-test, *P* = 0.041; *n* = 6; Fig. 5e). Similarly, depolarization of NA (−) neurons was elicited by bath application of the A_2A_R agonist CGS (1 μM; 3.30 ± 0.66 mV; paired *t*-test, *P* = 0.025; *n* = 5; Fig. 5d,e); this CGS-induced depolarization continued in the presence of TTX (1 μM; 2.69 ± 0.94 mV; paired *t*-test, *P* = 0.043; *n* = 4; Fig. 5e). These experiments reinforce our hypothesis of a direct action of ADO on NA (−) neurons through A_2A_R activation.

### Glucose promotes NA (−) neuron activation via two distinct pathways

**Figure 6:**
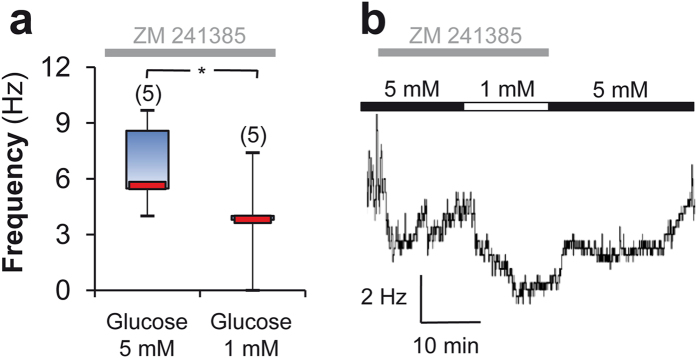
A_2A_R blockade does not prevent the glucose sensitivity of VLPO NA (−) neurons. (**a**) Box plots of NA (−) neurons recorded in ZM 241385 at 5 and 1 mM glucose. The median is plotted as the central red bar of the box, the upper and lower quartiles are the edges of the box and the whiskers show the extremes of the distribution. Sample sizes are indicated in parentheses. *P < 0.05; paired *t*-test. (**b**) Example of a firing frequency kinetic from a NA (−) neuron presented in (a). Note that 2 steps mark the return to the base-line level for the ZM 241385 first and for the glucose effect as previously reported^21^.

Previously, we established that glucose specifically increases the excitability of VLPO sleep-promoting neurons requiring the intraneuronal metabolization of glucose to ATP, which in turn inhibits ATP-sensitive potassium (K_ATP_) channels and consequently depolarizes the cell^21^. The present study confirms these results and demonstrates that glucose elicits astrocytic ADO release, which promotes *in fine* the excitation of sleep-promoting VLPO neurons. To confirm the involvement of these two different pathways, we assessed whether the excitatory effect of glucose on VLPO NA (−) neurons persists in the presence of ZM 241385 (10 μM) in order to block the adenosinergic pathway. NA (−) neurons that still displayed a sustained firing frequency under this treatment exhibited a significant decrease in their firing rate, when the glucose concentration was switched from 5 to 1 mM (corresponding to a drop in frequency from 6.68 ± 1.06 Hz to 3.80 ± 1.17 Hz; paired *t*-test, *P* < 0.05; *n* = 5; Fig. 6).

**Figure 7:**
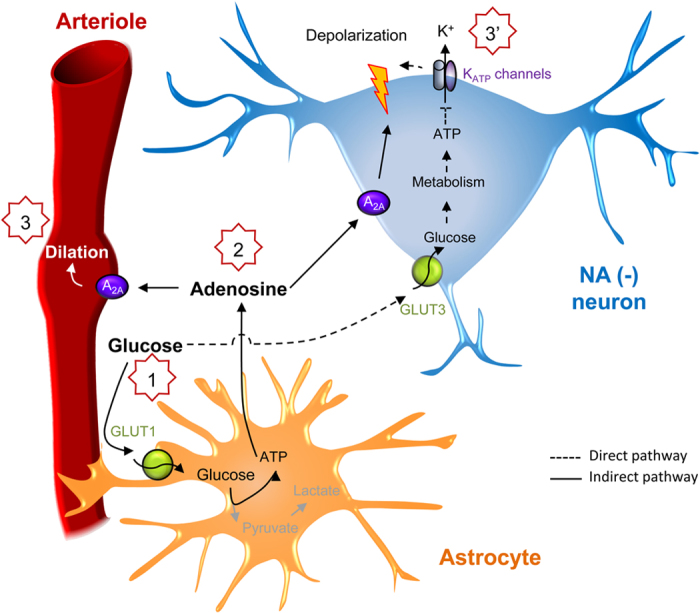
Schematic representation of the direct and indirect pathways mediated by glucose in the VLPO. Blood glucose passes into the brain parenchyma (1) and is either taken up by astrocytes (indirect pathway) or neurons (direct pathway). In the indirect pathway, glucose catabolism causes an increase in ATP production followed by an increase in the extracellular adenosine concentration (2). Subsequently, activation of A_2A_R induces vasodilation (3) and promotes an increase in the firing frequency of NA (−) neurons (3′). In the direct pathway, glucose catabolism by neurons causes an increase in ATP production and the closure of K_ATP_ channels, which strengthens the depolarization of NA (−) neurons induced by adenosine (3′).

Altogether, these results indicate that the increased extracellular glucose concentration leads to neuronal depolarization of VLPO NA (−) neurons by two distinct pathways: i) through a direct uptake and metabolization of glucose by NA (−) neurons^21^; and ii) likely through an indirect pathway involving glucose uptake by astrocytes, subsequent adenosine release and *in fine* activation of A_2A_R on NA (−) neurons (Fig. 7).

## Discussion

Here, we demonstrate for the first time that an increase in extracellular glucose concentration induces ADO release from an astrocytic source, and that ADO accumulates in the VLPO in a circadian-dependent manner. In addition, we have established that this nucleoside is able to activate VLPO sleep-promoting neurons and to dilate nearby blood vessels, both of which imply activation by A_2A_R. This mechanism could constitute an auto-excitatory loop within the VLPO that functions to sustain sleep-promoting neuronal activity under high-glucose supply, consequently promoting SWS. Taken together, our results support a novel ADO-mediated glucose pathway that may participate in the homeostatic sleep drive.

### Glucose-induced vasodilation implies glucose uptake, metabolization and ADO release by astrocytes

Glucose uptake in astrocytes *vs.* neurons is still strongly debated in the literature^45^,^46^,^47^. Here, we show that glucose-induced vasodilation in the VLPO requires cellular glucose uptake, since a non-specific GLUT transporter (cytochalasin B) completely prevented glucose-induced vasodilatation. Vasodilatations were also significantly impaired in the presence of phloretin, a GLUT1 transporter inhibitor that is the main glucose transporter expressed on astrocytes^31^,^48^. Nevertheless, we observed a residual vasodilatory response under these treatments. This could imply glucose uptake by neurons that predominantly express GLUT3 and result in the subsequent release of a vasodilatory compound; alternatively, this response could rely on other forms of GLUT expressed by astrocytes. These pharmacological experiments suggest that glucose uptake by astrocytic GLUT1 is a crucial step in the cerebral energy apparatus leading to vasodilation in the VLPO. This is consistent with previous studies showing that the end-feet of astrocytes completely surround blood vessels^49^, and that the majority of glucose entering the brain is subsequently taken up by astrocytes via GLUT1^48^.

Our results demonstrate that glucose-induced vasodilation requires the metabolization of glucose by hexokinase since bromopyruvic acid, a hexokinase inhibitor, significantly blocked dilations. However, treatment with alloxan, a glucokinase inhibitor, did not alter glucose-induced vasodilation. Based on the observations that glucokinase mRNA is only present in neurons^32^, and that glucose-induced vasodilation is not impaired by TTX or 4-CIN, we hypothesise that, under our conditions, glucose is preferentially metabolized in astrocytes. This result is in line with the previous observation that neurons have a lower glycolytic rate than astrocytes^50^, particularly because a key activator of glycolysis, the enzyme 6-phosphofructo-2-kinase/fructose-2,6-biphosphatase-3, is active only in astrocytes^51^.

We also found that glucose-induced vasodilatation was significantly reduced following A_2A_ antagonist treatment, suggesting a role for ADO in the vasodilatory response. Using purine biosensors, we revealed that an increase in extracellular glucose concentration elicits an extracellular increase in the amount of ADO in the VLPO, which is blocked following a glial toxin treatment. This result strengthens the hypothesis of a glial origin of ADO and supports previous findings indicating that extracellular levels of ADO are primarily regulated by astrocytes^52^,^53^,^54^,^55^. This finding is also in agreement with the previous observation that hypothalamic glia can respond to a rise in extracellular glucose with a large increase in glycolytic ATP production^56^, which is expected to lead to the glial release of ADO. Subsequently, ADO dilates nearby blood vessels and neurons. Finally, dose-response cuves reveal that glucose-induced vasodilation has an EC_50_ of 2.41 mM and should therefore be recruited at low glucose concentrations, prior to the direct catabolism of glucose in sleep-promoting neurons exhibiting a higher EC_50_ of 4.06 mM^21^.

### The increase in extracellular ADO concentration depends on the VLPO’s circadian rhythms

Extracellular ADO levels were previously reported to be sensitive to diurnal rhythms in the basal forebrain^57^ and the hippocampus^44^. Using purine biosensors, we reported that extracellular ADO is drastically increased in the VLPO at the circadian activity period ZT 1 as compared to ZT 12. Therefore, we hypothesise that the cumulative effect of glucose intake during the wake-active period should participate in the accumulation of extracellular ADO in the VLPO, resulting in the homeostatic sleep drive. Thus, glucose ingested during a meal at the end of the active period should have a greater hypnogenic effect relative to any earlier sugar ingestion during the day.

Potentially stable extracellular ADO levels have been reported by microdialysis measurements in the feline POAH/VLPO area during 6 h of sleep deprivation^58^. Interestingly, ADO levels decreased during sleep recovery, in support of our observation that basal ADO levels are lower at ZT 12 as compared to ZT 1. However, the explanation for why ADO increases during wakefulness, as well as with sleep deprivation in certain brain regions but not others, remains unknown.

Many factors determine the levels of extracellular ADO including exocytosis^35^,^59^,^60^, hemichannels^61^, transporters such as equilibrative nucleoside transporters (ENTs) and concentrative nucleoside transporters (CNTs)^62^, and degradative enzymatic activity resulting from a cascade of ectonucleotidases^63^. Further work is required to elucidate the molecular pathways underlying extracellular ADO release in the VLPO in response to increased glucose concentration, which is beyond the scope of the present study.

### A_2A_R-mediated activation of VLPO NA (−) neurons

*In vitro* studies performed in rats have demonstrated that ADO can either increase the excitability of VLPO neurons indirectly via presynaptic disinhibition^10^,^64^, or directly via A_2A_R activation. Indeed, an A_2A_R agonist has been reported to directly increase the firing rate of VLPO NA (−) neurons^8^, however the excitatory effect of ADO was shown to require A_1_R blockade^8^. Here, we have provided novel evidence in mice that ADO increases the firing rate of VLPO NA (−) neurons by direct neuronal A_2A_R activation and without A_1_R blockade. This result supports the hypothesis that A_2A_R-mediated activation of VLPO neurons contributes to the adenosinergic regulation of SWS^11^,^65^,^66^.

We determined in this study that ADO can exert important regulatory functions through the activation of vascular and neuronal ADO receptors, which supports observations in other structures where ADO has already been identified as an important mediator coupling cerebral blood flow to neuronal activation^9^,^67^. However, we cannot exclude that ADO might also bind astrocytic ADO receptors^68^ in an autoregulatory loop. Indeed, the ADO-evoked calcium response in acutely isolated astrocytes is reported to be coupled to the A_2B_ receptor^69^ and could also participate in neuronal excitability via the release of gliotransmitters. Activation of astrocytic A_2A_R that have been reported to decrease the uptake of glutamate^70^ could also be involved in glucose-induced VLPO neuronal activation. However, any astroglial contribution to glucose sensitivity would not be specific to NA (−), thus favouring the hypothesis of a selective expression of A_2A_R on sleep-promoting neurons.

## Conclusions

Our findings provide evidence for the direct uptake and metabolism of glucose by VLPO sleep-promoting neurons, and demonstrate that the increase in extracellular glucose concentration induces astrocytic-derived ADO release and neuronal A_2A_R activation. The novel metabolic pathway that we describe brings new emphasis to the central role of astrocytes in brain metabolism and neurovascular coupling processes, which are already known to have essential functions in capturing glucose from the circulation and coordinating neuronal firing with the local blood supply. These findings also define a key role for A_2A_R activation in the adenosinergic regulation of SWS. A better understanding of the role of glial cells in sleep regulation may provide new targets for treating brain dysfunctions, particularly those that are comorbid with sleep disorders.

## Materials and Methods

### Animals

All animal procedures were conducted in strict compliance with our institutional protocols and were approved by with the European Community Council Directive of 22 September 2010 (010/63/UE) and the local ethics committee (Comité d’éthique en matière d’expérimentation animale number 59, C2EA—59, ‘Paris Centre et Sud’). Accordingly, the number of animals in our study was kept to the necessary minimum. Male C57Bl6J mice (14–21 days old; Charles River, France) were used for all experiments. Mice arrived in the laboratory at least 1 week before experiments to acclimate to their new environment, and were housed in a temperature-controlled (21–25 °C) room under daylight conditions.

### Preparation of acute hypothalamic slices

Mice were rapidly decapitated and brains were quickly removed and placed into cold (∼4 °C) artificial cerebrospinal fluid (aCSF) containing (in mM): 130 NaCl; 5 KCl; 2.4 CaCl_2_; 20 NaHCO_3_; 1.25 KH_2_PO_4_; 1.3 MgSO_4_; 10 d-glucose; and 15 sucrose (pH = 7.35). Brains were constantly oxygenated with 95% O_2_–5% CO_2_. During slicing, 1 mM kynurenate was added to the aCSF. Coronal brain slices (300 μm thick) containing the VLPO were cut with a vibratome (VT2000S; Leica) and transferred to a constantly oxygenated (95% O_2_–5% CO_2_) holding chamber containing aCSF. Next, individual slices were placed in a submerged recording chamber maintained at 32 °C and perfused (1.5 ml/min) with oxygenated kynurenate-free aCSF. Blood vessels were visualised using infrared videomicroscopy, with Dodt gradient contrast optics (Luigs and Neumann) mounted on an upright microscope (Zeiss) equipped with a CDD camera (Hamamatsu).

### Drugs

To block neuronal synaptic transmission, the switch in glucose concentration was applied in the presence of tetrodotoxin (TTX; 1 μM; Latoxan) as well as 10 min after TTX application. To block NOS, slices were treated for at least 1 h with an irreversible inhibitor of constitutive nitric oxide synthase (nNOS) and a reversible inhibitor of inducible nitric oxide synthase (iNOS), Nω-Nitro-L-arginine (L-NNA; 100 μM; Sigma); alternatively, slices were treated with carboxy-PTIO, a specific nNOS inhibitor (30 μM). NO-trapping was performed by superfusing the slice with bovine haemoglobin (4 μM; Sigma). To block the production of prostaglandins, slices were preincubated for at least 30 min with aspirin (50 μM; Sigma), an inhibitor of the cyclooxygenase COX-1 isoform, or with the selective COX-2 inhibitor N-[2-(Cyclohexyloxy)-4-nitrophenyl]methanesulfonamide (NS-398; 10 μM; Sigma).

The lactate shuttle was blocked with α-Cyano-4-hydroxycinnamic acid (4-CIN; 500 μM; Sigma), a specific inhibitor of monocarboxylic transport; glucokinase was inhibited with 2,4,5,6(1H,3H)-Pyrimidinetetrone (Alloxan; 10 mM; Sigma); hexokinase was inhibited by 3-Bromo-2-oxopropionic acid (Bromopyruvic acid; 100 μM; Sigma); and cytochalasin B was used as a non-selective glucose transporter (GLUT) inhibitor (25 μM; Sigma) whereas β-(4-Hydroxyphenyl)-2,4,6-trihydroxypropiophenone (Phloretin; 10 μM; Sigma) was used as a selective GLUT1 inhibitor. A_1_ receptors were blocked with the selective antagonist 8-Cyclopentyl-1,3-dipropylxanthine (DPCPX; 10 μM; Sigma) and A_2A_ receptors by the selective antagonist ZM 241385 (4-(-2-[7-amino-2-{2-furyl}{1,2,4}triazolo{2,3-a} {1,3,5}triazin-5-yl-amino]ethyl)phenol; 100 μM; Sigma). All blockers were applied at least 5 min before the increase in glucose concentration. The thromboxane A_2_ receptor agonist 9,11-dideoxy-11a,9a-epoxymethanoprostaglandin F2α (U46619; 10 nM; Sigma) was used to preconstrict blood vessels. 9-β-D-Ribofuranosyladenine (Adenosine, ADO; 100 μM; Sigma) and the A_2A_ receptor agonist CGS-21680 hydrochloride hydrate (CGS; 1 μM; Sigma) were used to stimulate VLPO neurons. Glial metabolism was inhibited using fluoroacetate (FAC; 5 mM and 50 min of preincubation; Sigma).

### Vascular reactivity

Blood vessels located in the VLPO or in the supragranular layers of the somatosensory cortex (S1) that were within the focal plane and exhibited a well-defined luminal diameter (10–20 μm) were selected for vascular reactivity. Images of blood vessels were acquired every 15 s with the Media Cybernetics software. The eventual drift of the images during the recording time was corrected either on-line for the z-drift or off-line for the x and y axes using Image Pro Plus 7.0. Manual replacement of images to minimize the differences between two consecutive frames was performed using a subtraction tool from Image Pro Plus. In order to determine the location with the most movement, luminal diameters were quantified at different locations along the blood vessel using custom written routines running within IgorPro (Wavemetrics). Control baselines were determined for 5 min at the start of the recording, with temperature maintained at 32 ± 1 °C. Unresponsive blood vessels or vessels with unstable baselines were discarded from the analysis. Vessels were considered unstable when their diameter moved more than 5% during the control baseline test. Only one vessel per slice was recorded.

Since blood vessels in slice preparations lack intraluminal flow and pressure^71^,^72^, vasomotor movements were detected in vessels that had been preconstricted for 10 min with the thromboxane agonist U46619 (10 nM), which was applied throughout the whole experiment^72^. Only blood vessels presenting a reduction in diameter of at least 10% were kept for analyses. Vasoconstriction of blood vessels induced by U46619 followed an exponential progression over time. This exponential drifting contraction was fitted and then subtracted from the recordings using IgorPro. Vasomotor responses were subsequently expressed as percentages of the mean baseline diameter, 1 min before the switch in glucose concentration from 1 to 5 mM. The effects of pharmacological blocking were then analysed by performing statistical analyses: comparisons were made between the 1 min period before the increase in glucose concentration and the mean blood vessel diameter change occurring either at 19–20 minutes in 5 mM glucose (for the vasodilatory response) or 10–11 minutes (for vasoconstrictions). Subsequently, a 20-minute recovery period was recorded in 1 mM glucose.

### Purine biosensor recording

Purine biosensors (Sarissa Biomedical, Coventry, United Kingdom) were used to detect extracellular levels of either adenosine (ADO) or inosine (INO). Each biosensor included a platinum wire 50 μm in diameter and 0.5 mm in length, coated with a permselective layer and an enzymatic matrix surrounding the platinum electrode^42^,^43^. For the ADO biosensors, the enzymatic layer contained nucleoside phosphorylase, xanthine oxidase, and adenosine deaminase, whereas the INO biosensors lacked adenosine deaminase and were sensitive to adenosine. Amperometric measurements started when hydrogen peroxide oxidized the platinum wire to create an electrical signal proportional to the purine concentration. Measurements with adenosine biosensors gave a total purinergic concentration of adenosine and its metabolites, referred to as ADO’^42^,^73^. In contrast, INO sensors were used to measure the response to inosine and its metabolites. We thus obtained a specific ADO signal by subtracting the signal of INO sensors from ADO sensors; approximately 55% of the signal from the ADO/INO biosensor was specific to adenosine in our conditions. Before use, all biosensors were rehydrated in a buffer containing (in mM): 100 NaCl, 50 Tris, 20 glycerol, 1 MgCl_2_, and 1 CaCl_2._ All biosensor sets were calibrated at the beginning and end of each experiment with 10 μM adenosine or inosine. The ADO and INO recordings were performed after a 30 min stabilization period. The sensors were inserted sequentially during a 3-minute period in the regions of interest in aCSF containing the following concentrations of glucose: first 1 mM, followed by 5 mM, and again 1 mM. Each recording at a different concentration was separated by 20 min. Biosensors were placed in contact with the surface of the slice by micromanipulators, which was visually confirmed as a slight deformation on its surface, indicating that the electrode was properly embedded. During the experiment, microelectrode biosensors were polarized at +500 mV versus Ag/AgCl. Purine concentrations were assessed by subtracting the electrical signal of the sensor when inserted into the region of interest from the mean of the electrical signals measured 30 s before and after the insertion. Biosensor signals were acquired with an amperometric detector (AMU 110; Radiometer Analytical, Villeurbanne, France) using an acquisition board (Digidata 1322A; Molecular Devices) attached to a computer running the pCLAMP 9.2 software package (Molecular Devices). Analyses were performed using the Clampfit (Molecular Devices), SigmaPlot (Systat) and Python software programs.

### Electrophysiological recordings

Electrophysiological experiments were performed at 32 ± 1 °C using a MultiClamp700B (Axon Instruments) amplifier connected to an acquisition board (Digidata 1440; Axon Instruments), filtered at 5 kHz, digitized at 50 kHz and recorded to a computer running pCLAMP9.2 software (Axon Instruments). Data were analysed off-line using the Clampfit software (Molecular Devices).

#### Extracellular recordings in loose cell-attached configuration

Loose cell-attached patch-clamp recordings were performed to monitor the stable spontaneous firing activity of VLPO neurons for long periods of time, as a prerequisite for completing the pharmacological experiments. Infrared videomicroscopy was used to visually select VLPO neurons. Loose-cell attached recordings were performed from the soma with patch micropipettes (3–6 MΩ) pulled from borosilicate glass capillary tubes (1.5 mm o.d., 0.86 mm i.d.; Harvard Apparatus, France) on a horizontal puller (Model P-1000; Sutter Instrument, Novato, CA, USA). Micropipettes were filled with oxygenated aCSF and fixed to an electric Microdrive (Luigs and Neumann, Germany). The micropipette was placed in contact with the soma of a selected neuron under visual control. During recordings, a seal resistance of 10–15 MΩ was maintained to avoid damage or mechanical stimulation to the cell.

#### Patch-clamp recordings in whole-cell configuration

Current clamp recordings were made without series resistance compensation. Patch-clamp pipettes (3–6 MΩ) were filled with the following (in mM): 144 K-gluconate; 3 MgCl_2_; 0.2 EGTA; 10 HEPES; 1.25 ATP (pH 7.2; 285–295 mOsm). The liquid junction potential of the patch pipette and the perfused extracellular solution were 11 mV and were not applied to the data. Data were rejected if the access resistance increased over 25 MOhm during a recording or changed by more than 25% during the recording.

### Statistical analyses

Data were expressed as mean ± standard error of the mean (SEM) and represented as a box plot, where the median is plotted as the central bar of the box, the upper and lower quartiles are the edges of the box, and the whiskers show the extremes of the distribution. The statistical significance of the data was assessed using either parametric tests (paired *t*-test and Student’s *t*-test) or non-parametric tests (Wilcoxon test and Mann-Whitney *U*-test), depending on whether the values from the considered data set followed a normal distribution or not.

## Additional Information

**How to cite this article**: Scharbarg, E. *et al.* Astrocyte-derived adenosine is central to the hypnogenic effect of glucose. *Sci. Rep.* **6**, 19107; doi: 10.1038/srep19107 (2016).

## Supplementary Material

Supplementary Video 1

Supplementary Information
