# Supplementary material for: Astrocyte-derived adenosine is central to the hypnogenic effect of glucose
Source: Sci Rep. 2016 Jan 12;6:19107. doi: 10.1038/srep19107 (PMC4709579; doi:10.1038/srep19107)
Supplement: Supplementary Information [file srep19107-s2.pdf]

Astrocyte-derived adenosine is central to the hypnogenic effect of glucose

Emeric Scharbarg[1,2], Marion Daenens[1,2], Frédéric Lemaître[3,4], Hélène Geoffroy[1,2], Manon Guille-Collignon[3,4], Thierry Gallopin[1,2] & Armelle Rancillac[1,2]*†

Supplemental Data

Video 1: Extracellular glucose increased concentration reversibly dilates intraparenchymal arterioles within the VLPO. Vascular response of a blood vessel that preconstrict under U46619 (10 nM) and then dilates in high glucose concentration (from 1 to 5 mM glucose; 20 min). Note the progressive come back to the baseline when returned to 1 mM glucose perfusion. Frame scan images: 15 s/frame.
